# Proteomic Analyses of Human Sperm Cells: Understanding the Role of Proteins and Molecular Pathways Affecting Male Reproductive Health

**DOI:** 10.3390/ijms21051621

**Published:** 2020-02-27

**Authors:** Ashok Agarwal, Manesh Kumar Panner Selvam, Saradha Baskaran

**Affiliations:** American Center for Reproductive Medicine, Cleveland Clinic, Cleveland, OH 44195, USA; pannerm@ccf.org (M.K.P.S.); saradhabaskaran@gmail.com (S.B.)

**Keywords:** sperm, proteomics, male infertility, bioinformatics, molecular pathways

## Abstract

Human sperm proteomics research has gained increasing attention lately, which provides complete information about the functional state of the spermatozoa. Changes in the sperm proteome are evident in several male infertility associated conditions. Global proteomic tools, such as liquid chromatography tandem mass spectrometry and matrix-assisted laser desorption/ionization time-of-flight, are used to profile the sperm proteins to identify the molecular pathways that are defective in infertile men. This review discusses the use of proteomic techniques to analyze the spermatozoa proteome. It also highlights the general steps involved in global proteomic approaches including bioinformatic analysis of the sperm proteomic data. Also, we have presented the findings of major proteomic studies and possible biomarkers in the diagnosis and therapeutics of male infertility. Extensive research on sperm proteome will help in understanding the role of fertility associated sperm proteins. Validation of the sperm proteins as biomarkers in different male infertility conditions may aid the physician in better clinical management.

## 1. Introduction

Spermatozoa are matured motile cells that are products of spermatogenesis. A healthy man produces between 20 to 240 million sperms per day [1]. Spermatogenesis is a series of cellular events that supports the production of sperm every day, from puberty onwards. Disturbance in the molecular events related to testicular spermatogenesis or post-testicular maturation may result in infertility. Infertility is defined as the inability to conceive after 12 months of regular, unprotected intercourse [2]. According to the World Health Organization (WHO), nearly 190 million people are infertile worldwide [3,4], and among the infertile couples, male factors contribute to 50% of cases [5]. In general, male infertility is evaluated based on semen analysis, which is considered as the corner stone of male infertility testing. Semen analysis provides information about sperm concentration, motility, morphology and vitality, and fertile men are distinguished from infertile men based on the 5th edition reference values established by the WHO laboratory manual [6]. However, the standard semen analysis fails to predict the fertility potential in men [7]. Advanced sperm function tests such as oxidation-reduction potential (ORP) [8] and terminal deoxynucleotidyl transferase dUTP nick end labeling (TUNEL) assay or sperm chromatin structure assay (SCSA) measure seminal oxidative stress and sperm DNA fragmentation (SDF), respectively [9,10], which can cause fertilization failure or male infertility [11,12]. However, these laboratory tests still fail to explain the underlying mechanisms at a subcellular level of spermatozoa that are associated with male infertility.

Currently, the focus has shifted towards investigating the molecular factors associated with spermatozoa that could adversely affect the fertilization process, with special attention given to the proteome of ejaculated spermatozoa [9,13,14,15]. Several studies have used the global proteomic approach in sperm cells to explain the molecular mechanisms regulating male reproductive health [16,17,18,19]. Sperm proteomics helps in understanding the cellular pathways, post-translational modifications and protein-protein interactions associated with normal gametogenesis and the role of proteins in the fertilization process.

Proteomic data enables better understanding of sperm biochemistry and provides information that aids in improving reproductive outcomes in infertility patients. For example, mitochondrial proteome reflects the functionality of mitochondria that is directly correlated with motility of sperm in asthenozoospermic patients [20] and oxidative stress in infertile men with varicocele [18]. Furthermore, functional analysis of sperm proteins using the bioinformatics approach serves as a promising tool in the identification of potential diagnostic and therapeutic biomarkers for the management of male infertility.

Revolution in the field of omics and availability of advanced proteomic tools have increased the knowledge and understanding of the causes of male infertility. This review provides a brief overview on proteomic techniques used to analyze sperm proteome. It also highlights the general steps involved in global proteomic approaches, including bioinformatic analysis of the proteomic data. Furthermore, proteomic-based studies in human sperm are discussed in detail, along with the potential role of biomarkers in the prognosis and diagnosis of male infertility.

## 2. Proteomics Overview

### 2.1. Process and Techniques

The profiling of proteins extracted from a tissue or cell is termed proteomics. Shotgun or bottom-up proteomics are common proteomic approaches to detect the proteins (>1000) in a very short period of time. Sperm proteins are detected using both conventional and advanced proteomic techniques. Two-dimensional (2D) gel electrophoresis is the most commonly used technique to separate sperm proteins based on the isoelectric focusing property and molecular weight of peptides. The 2D-gel electrophoresis coupled with matrix-assisted laser desorption/ionization time-of-flight (MALDI-TOF) technique was used to identify 98 distinct proteins in the human spermatozoa [21]. A modified version of the 2D-gel electrophoresis technique, known as difference gel electrophoresis (DIGE), is used to identify differentially expressed proteins (DEPs) with a minimum error of <10% [22]. Based on the intensity of the different staining dyes (Cy3 and Cy5), the expression of the DEPs is determined on the same gel using an automated image analysis software, such as Typhoon Trio Imager (GE Healthcare) [23]. Advanced proteomic techniques include analysis of sperm proteins using MALDI-TOF and liquid chromatography-tandem mass spectrometry (LC-MS/MS). These instruments can detect the maximum number of proteins, even in samples that are of lesser concentration. Using the in-gel digestion based LC-MS/MS approach, Johnston et al. identified 1760 sperm proteins [24]. Later on, several other studies also employed the LC-MS/MS-based proteomic profiling of spermatozoa in male infertility cases [9,20,25,26,27]. In general, comparative protein analysis involves incorporation of stable isotopes for labeling the peptides. Such techniques include tandem mass tags (TMTs), isobaric tag for relative and absolute quantitation (iTRAQ) labelling, stable isotope labeling by amino acids in cell culture (SILAC) and isotope-coded affinity tag (ICAT). Among these techniques, iTRAQ labelling is widely used in sperm proteomics [27,28]. Recently, an alternative label-free technique was developed to profile the proteins in sperm cells. Liu et al. used label-free proteomic approach to profile the proteins in the spermatozoa of asthenozoospermic obese men [29]. Similarly, Moscatelli et al. also used the same approach to identify the protein involved in the bioenergetics pathways affecting sperm motility [30].

### 2.2. Proteomic Analysis Using LC-MS/MS

Semen contains cellular (spermatozoa) and non-cellular (seminal plasma) components, the latter is enriched with proteins, thereby representing a target for seminal plasma proteomics and a putative source of markers [31,32]. Other than spermatozoa, semen also contains round cells, which are of two types: Spermatogenic and non-spermatogenic round cells. Few studies have proposed that the use of sperm with round cells in the protein extraction process may contaminate sperm proteome. Separation of round cells and leukocytes are carried out using gradient centrifugation method and the pure fraction of the spermatozoa are used for proteomic analysis [21,33,34,35,36,37]. Recently, Panner Selvam et al. investigated the interference of round cell proteins in the proteome of sperm and their effect on biological pathways associated with sperm function [38,39]. The influence of non-spermatogenic round cell proteins was found to be very negligible or insignificant when compared to that of the sperm proteome [38]. Furthermore, the presence of these round cells and leukocyte proteins did not interfere in the molecular pathways associated with sperm function [39]. However, while investigating the physiological functions of spermatozoa such as hyperactivation, capacitation and acrosome reaction, the use of pure fractions of normal sperm isolated using double gradient centrifugation or swim-up techniques has been reported [40,41].

**Figure 1:**
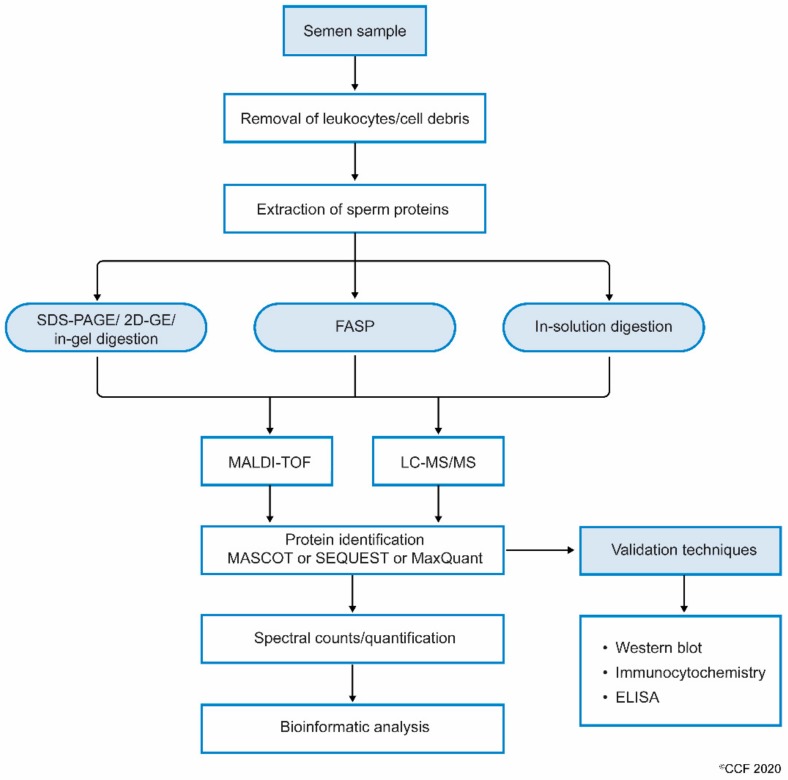
Workflow involving the processing of semen samples for sperm proteomics.

When processing the sperm samples, the spermatozoa are first separated from semen or seminal fluid by centrifugation. The isolated sperm are washed 3 to 4 times with phosphate-buffered saline (PBS) to remove the remnants of seminal plasma. The sperm pellet is then mixed with lysis buffer (often radioimmunoprecipitation assay (RIPA) solution with SDS) and left overnight. This results in the complete lysis of spermatozoa [38]. Sperm proteins are also extracted by sonicating the sperm cells suspended in an isotonic medium. Extracted sperm proteins are checked for their purity and concentration, and then subjected to electrophoresis (either one-dimensional or 2D-gel). Other methods, such as filter aided sample preparation (FASP) and in-solution, are also used to prepare samples for proteomic analysis. Samples subjected to FASP provide comparatively more proteome coverage, compared to samples processed using in-gel and in-solution digestion for mass spectrometry-based proteomics [42]. Wang et al. used the FASP coupled MS/MS method to identify N-glycosylated proteins and glycosylation sites in human sperm [43]. The protein extract can either be subjected to (i) SDS PAGE and in-gel digestion, or (ii) in-solution digestion, or (iii) FASP (Figure 1).

MS detects the peptides and proteins using an unbiased approach [44]. The proteins are indirectly inferred from the peptide with a very low false discovery rate, based on their mass/charge ratio (*m*/*z*). In general, the results of proteomics experiments contain 1–5% false positive protein identifications based on the inferred peptides using MS. To identify post-translational modification, such as acetylation, methylation and phosphorylation in the sperm proteome, enrichment protocols are recommended. In addition, LC-MS/MS is used to reduce complexity of the peptide mixture that is obtained after the digestion of the proteins. Other techniques such as MALDI-TOF and SELDI-TOF (surface-enhanced laser desorption/ionization time-of-flight) are also successfully used to detect sperm proteins [45,46].

Next, the complete scan of peptides detected by the MS are initially compared with the global database consisting of previously annotated and sequenced genes corresponding to respective proteins. At the least, masses of three peptide fragments should match with the corresponding homologous peptide masses in the protein database. Computational software such as SEQUEST, Mascot, MaxQuant and X!-Tandem, that operate using different algorithms, then display the complete list of proteins [47]. In addition, the proteins are categorized as DEPs based on spectral counts (SC) and abundance of each protein. These DEPs are used in the downstream bioinformatic analysis to understand the role of proteins in molecular pathways [48]. Gene ontology (GO) analysis provides additional information such as localization and functional annotation of proteins. Freely available bioinformatics tools such as STRING (Search Tool for the Retrieval of Interacting Genes/Proteins) are used to display the interaction between proteins [49]. In addition, commercially available sophisticated software such as Ingenuity Pathway Analysis (IPA) and Metacore™ are used to obtain a complete picture of the interactions between the proteins and the transcriptional factors regulating their expression [50]. Furthermore, careful interpretation of the bioinformatics results is required for sperm, as these tools have been developed for somatic cells.

## 3. Proteomic Analysis of Human Spermatozoa

Spermatozoa are regarded as an excellent candidate for proteomic studies since (a) they can be easily purified at high concentrations from semen, and (b) they do not generate new proteins as they are transcriptionally and translationally quiescent, which in turn reduces the complexity of proteomic profiling of spermatozoa [51,52]. Therefore, analyzing and cataloging sperm proteome could provide a deeper understanding of the machineries and their significance in key processes associated with the fertilizing ability of spermatozoa.

Naaby-Hansen and colleagues, the pioneers of sperm proteomics, provided the first comprehensive human sperm protein database of 1400 2D spots [53]. Although earlier proteomic studies were focused on identifying specific sperm surface proteins [54], the first detailed report on human spermatozoa proteome was published in 2005 using 1D-SDS-PAGE coupled with LC-MS/MS analysis [24]. The study identified 1760 distinct proteins in human spermatozoa and provided the first definite evidence that all 27 proteins constituting the 26S proteasome are present in the sperm [24]. Although the study provided the functional composition of proteins in soluble and insoluble fractions of spermatozoa, no specific list of proteins was presented. In 2006, characterization of proteins extracted from human spermatozoa by 2-DE, and subsequently subjected to MALDI-TOF MS analysis resulted in the identification of 98 distinct proteins [21]. The functional distribution of these proteins was reported to be energy production (23%), transcription, protein synthesis, transport, folding and turnover (23%), cell cycle, apoptosis and oxidative stress (10%), signal transduction (8%), cytoskeleton, flagella and cell movement (10%), cell recognition (7%) and metabolism (6%) [21]. In 2007, Baker et al. identified 1053 proteins using LC-MS/MS analysis and the protein inventory included nicotinamide adenine dinucleotide phosphate oxidase (NOX), and its homolog, dual oxidase 2 (DUOX2), and different classes of receptors that are potential regulators of sperm function [55]. Later, Gilany et al. reported a collection of 1300 proteins involved in various metabolic pathways [56]. With the advancements in the proteomic platform, Wang et al. identified 4675 human sperm proteins, out of which 227 were testis-specific [57].

Initially, the proteomic studies were largely focused on whole sperm proteome, however, subcellular proteomics have gained substantial attention as it provides explicit information on sperm protein content as well as their precise localization. In the subcellular proteomic approach, the spermatozoa are separated into fractions, such as head, tail or membrane, by differential centrifugation and each fraction is then subjected to proteomic analysis [37,58,59,60]. Assessment of isolated tail and membrane fractions of spermatozoa led to the elucidation of metabolic enzymes vital for motility [37] and proteins putatively involved in the mediation of sperm-oocyte interaction [58], respectively. In addition, findings of the study conducted by Amaral et al. revealed a surprisingly high number of peroxisomal protein in sperm that is thought to be devoid of peroxisomes [37]. Baker et al. analyzed the proteome of sperm head and tail fractions, and reported 900 proteins in sperm tail, 700 proteins in sperm head, and 159 overlapping proteins were found in both of the subcellular fractions [60]. De Mateo et al. isolated sperm nuclei and identified 403 proteins by LC-MS/MS analysis [59]. Furthermore, de Mateo et al. were the first to report correlation between proteomics, DNA integrity and protamine content [61]. A comprehensive analysis of whole and subcellular sperm proteomic studies conducted by Amaral et al. involving 30 different studies, identified and cataloged 6198 proteins [62]. The proteins were categorized based on their involvement in various functional pathways including metabolism, cell cycle, apoptosis, membrane trafficking, RNA metabolism and post-translational protein modifications [62].

## 4. Clinical Implications of Sperm Proteomics

### 4.1. Protein Profiling in Male Infertility

Over the past few decades, sperm proteomics have been investigated by reproductive scientists around the world, however, research on the implications of sperm proteomics in male infertility evaluation has gained tremendous momentum in the last 10 years. Clinical/comparative proteomics have paved the way for identifying DEPs related to various clinical scenarios associated with male infertility (Table 1).

#### 4.1.1. Protein Profiling in Asthenozoospermia

Asthenozoospermia is a common cause of male infertility, characterized by reduced sperm motility. Several comparative sperm proteomic studies have been conducted to delineate the proteins and associated pathways implicated in the molecular pathophysiology of asthenozoospermia [63,64,65,66]. The reduction of sperm motility has been recognized as a consequence of several factors, such as energy metabolism dysfunction, structural defects in sperm-tail protein components and differential expression of proteins involved in sperm motility such as cytochrome c oxidase subunit 6B (COX6B), outer dense fiber 2 (ODF) and tubulin beta 2B (TUBB2B) [20]. Saraswat et al. identified altered expression of proteins (see Table 1) associated with axoneme activation and focal adhesion assembly, glycolysis, gluconeogenesis, cellular response to stress and nucleosome assembly [64]. Comparative analysis of sperm proteome of normozoospermic and asthenozoospermic subjects by two-dimensional PAGE MALDI MS/MS resulted in the identification of eight proteins with altered expression [67]. These DEPs were disseminated into three functional groups namely: ‘Energy and metabolism’ (triose-phosphate isomerase; TPIS, testis-specific glycerol kinase 2; GKP2), and succinyl-CoA:3-ketoacid co-enzyme A transferase 1, mitochondrial precursor; OXCT1); ‘movement and organization’ (tubulin beta 2C; TUBB2C and tektin 1; TEKT1) and ‘protein turnover, folding and stress response’ (proteasome alpha 3 subunit; PSMA3 and heat shock-related 70 kDa protein 2; HSPA2). Other proteomic studies have also noted that the majority of the identified DEPs fall into similar categories of proteins [68,69]. Furthermore, sperm proteomic analysis in asthenozoospermic subjects has revealed differential expression of some components of the proteasome complex, indicating the significance of the proteasome complex in sperm motility [67,68,69]. Comparative proteomic analysis on sperm tail fractions of the samples from asthenozoospermia and normozoospermia resulted in the identification of fourteen DEPs that are associated with sperm functions [70]. Nowicka-Bauer et al. correlated the DEPs identified in asthenozoospermia with the functional status of mitochondria, which indicated the possible role of sperm mitochondrial dysfunction and oxidative stress in the molecular etiology of asthenozoospermia [65]. Based on these reports, we speculate that oxidative stress impairs mitochondrial function and energy metabolism, which in turn alters the expression of structural proteins leading to reduced sperm motility in asthenozoospermic patients. Furthermore, findings of another recent sperm proteomic study revealed decreased expression of TEX40 and ATP6V0A2 proteins related to calcium ion entry and acrosomal acidification in asthenozoospermic men [66]. The downregulation of TEX40 results in fewer entry of calcium ions into the sperm and decreased expression of ATP6V0A2 leads to acrosomal de-acidification, which in turn diminishes sperm motility in asthenozoospermic males.

#### 4.1.2. Protein Profiling in Azoospermia

About 15% of infertile men have azoospermia, a condition associated with lack of measurable level of sperm in the semen [71]. There are two major forms of azoospermia, namely, obstructive azoospermia (OA) and non-obstructive azoospermia (NOA). OA occurs due to physical obstruction in the male reproductive tract preventing the entry of sperm into ejaculate. On the other hand, NOA can be classified into subtypes of testicular failure, which includes maturation arrest (MA) and failure of germ cell maturation, and Sertoli cell only syndrome (SCOS) (i.e., presence of only Sertoli cells in the seminiferous tubules with complete absence of spermatogonial cells) [71,72]. There are no sperm proteomic studies on azoospermia as it is associated with absence of sperm in the ejaculate. However, a few proteomic studies have been conducted in the seminal plasma and testicular tissues of azoospermic subjects to identify potential biomarkers [73,74,75]. A proteomic study conducted by Drabovich et al. on the seminal plasma from men with normal spermatogenesis and azoospermia, revealed extracellular matrix protein 1 (ECM1) as a biomarker to differentiate OA from normal spermatogenesis, and OA from NOA with a cutoff level of 2.3 µg/mL [73]. Furthermore, a differential expression of testis-expressed protein 101 (TEX101) in distinct NOA subtypes have been reported [73]. Proteomic analysis of testicular tissue samples from OA and NOA identified differential expression of Y-box protein 1 (YBX1), lactate dehydrogenase C (LDHC), chaperonin containing TCP1 subunit 7 (CCT7), and matrin-3 [75]. The study also demonstrated that spliceosome, cell cycle, and proteasome proteins along with energy and metabolism related proteins were drastically suppressed in SCOS, while altered to a lesser extent in MA [75].

#### 4.1.3. Protein Profiling in Oligoasthenozoospermia

Oligoasthenozoospermia is a combination of oligozoospermia and asthenozoospermia, characterized by reduced sperm concentration and motility. Review of available literature reveals very few proteomic studies on the seminal plasma, and no studies in spermatozoa of oligoasthenozoospermic subjects [26,76,77]. Proteomic characterization of seminal plasma carried out by Herwig et al. explicated the role of oxidative stress in the underlining mechanism, and identified a panel of 46 proteins that are involved in the etiology of oligoasthenoteratozoospermia due to oxidative stress [76]. Comparative proteomic analysis of oligoasthenozoospermic and normozospermic seminal plasma identified DEPs involved in multiple biological functions, such as binding activity (lactotransferrin, LTF; Prolactin-induced protein, PIP; extracellular matrix protein 1, ECM1), transporter activity (human epididymis-specific *protein* 1, HE1; Prostaglandin D_2_ synthase, PTGDS), immune activity (CD177), and hydrolase activity (prostate-specific antigen) [77].

#### 4.1.4. Protein Profiling in Globozoospermia

Globozoospermia is a rare and severe form of teratozoospermia which accounts for <0.1% of male infertility [78]. It is characterized by round headed spermatozoa lacking an acrosome with deranged nuclear membrane and midpiece defects. Genetic studies have revealed that mutations or deletions in three genes namely, SPATA16, PICK1 and DPY19L2, are responsible for globozoospermia [79]. Proteomic studies have enabled a better understanding of round-headed spermatogenesis and the proteins implicated in its underlying pathophysiology. Liao et al. conducted a comparative analysis of proteome of normal and round-headed spermatozoa using 2D fluorescence DIGE coupled with MS/MS [80]. About 35 protein spots showed differential expression, with nine proteins upregulated and 26 proteins downregulated in round-headed spermatozoa, when compared to normal spermatozoa. These differentially expressed proteins were identified to play an important role in a variety of cellular processes including, spermatogenesis, cell skeleton, metabolism and motility [80]. Acrosome biogenesis, a vital step in sperm differentiation, depends on the precise formation of golgi-derived proacrosomal vesicles and concurrent modifications in the nuclear envelope. A set of proteins collectively known as perinuclear theca (PT), which have been implicated in acrosome development are reported to be significantly decreased in globozoospermic patients [81].

### 4.2. Proteomic Profiling in Infertility-Related Conditions and Diseases

#### 4.2.1. Protein Profiling in Varicocele

**Figure 2:**
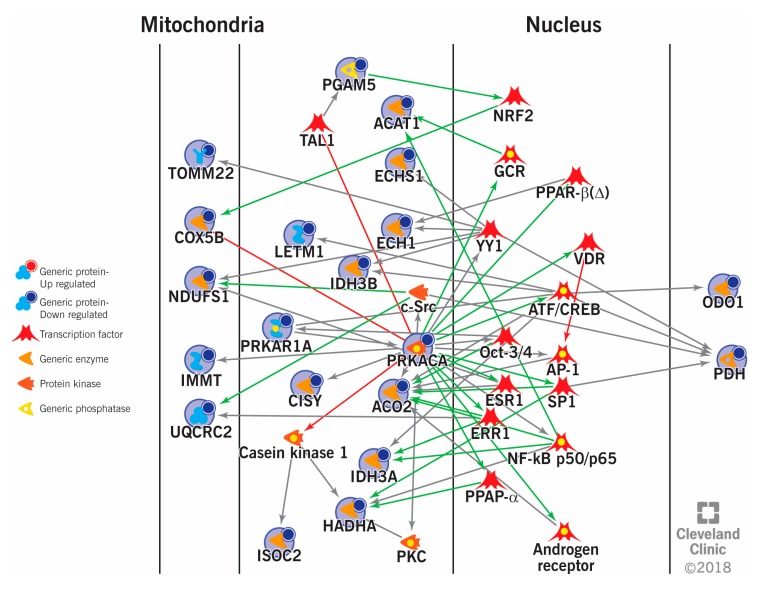
Interaction between the differentially expressed proteins and transcriptional factors in varicocele patients with mitochondrial dysfunc­tion. TOMM22: mitochondrial import receptor subunit TOM22 homolog, COX5B: cytochrome c oxidase subunit 5B, mitochondrial, NDUFS1: NADH-ubiquinone oxidoreductase 75 kDa subunit, mitochondrial, IMMT: NADH-ubiquinone oxidoreductase 75 kDa subunit, mitochondrial, UQCRC2: cytochrome b-c1 complex subunit 2, mitochondrial, PGAM5: serine/threonine-protein phosphatase PGAM5, mitochondrial, TAL1: T-cell acute lymphocytic leukemia protein 1, ACAT1: acetyl-CoA acetyltransferase, mitochondrial, ECHS1: enoyl-CoA hydratase, mitochondrial, ECH1: delta(3,5)-delta(2,4)-dienoyl-CoA isomerase, mitochondrial, LETM1: mitochondrial proton/calcium exchanger protein, IDH3B: isocitrate dehydro­genase [NAD] subunit beta, mitochondrial, c-Src: proto-oncogene tyrosine-protein kinase Src, PRKAR1A: cAMP-dependent protein kinase type I-alpha regulatory subunit, PRKACA: cAMP-dependent protein kinase catalytic subunit alpha, CISY: citrate synthase, mitochondrial, ACO2: aconitate hydratase, mitochondrial, IDH3A: isocitrate dehydrogenase [NAD] subunit alpha, mitochondrial, HADHA: trifunctional enzyme subunit alpha, mitochondrial, ISOC2: isochorismatase domain-containing protein 2, PKC: protein kinase C, NRF2: nuclear factor erythroid 2 (NFE2)-related factor 2, GCR: glucocorticoid receptor, PPAR-β (Δ): peroxisome proliferator-activated receptor beta or delta, YY1: Yin Yang 1, VDR: vitamin D receptor, ATF: activating transcription factor, CREB: cAMP response element binding, OCT-3/4: octamer-binding transcription factor 3/4, AP-1: activator protein 1, SP1: specificity protein 1, ESR1: estrogen receptor 1, ERR1: steroid hormone receptor ERR1, NF-κB: nuclear factor kappa-light-chain-enhancer of activated B cells.

Varicocele, an abnormal dilation of pampiniform plexus, is one the most common and correctable causes of male infertility [82]. Since it is more prevalent in men of the reproductive age group, early diagnosis and management is crucial. Analysis of sperm proteomic profile facilitates identification of proteins with altered expression related to male infertility. Hosseinifar et al. were the first to compare the proteomic differences in the spermatozoa of men with and without varicocele [83]. The study identified 15 DEPs that mainly included heat shock proteins (HSPs), mitochondrial proteins, and cytoskeleton proteins [83]. Chang et al. also reported an increased expression of HSPs (HSP70 and 90) in varicocele subjects, compared to that of control men [84]. Comparative analysis of the sperm proteomic profile between unilateral varicocele subjects and fertile men, revealed 369 DEPs with an overexpression of 114 proteins and underexpression of 97 proteins in the unilateral varicocele group. Of the 369 DEPs, 29 proteins were involved in spermatogenesis and other key reproductive events such as sperm maturation, motility, capacitation, acrosome reaction and fertilization. Furthermore, functional annotation revealed that small molecule biochemistry and post-translation modification proteins are mostly affected by unilateral varicocele [85]. Subsequently, in another study, Agarwal et al. reported for the first time, the differences in the expression profile of sperm proteins between infertile men with unilateral and bilateral varicocele [86]. Out of 253 DEPs, 21 proteins were involved in key reproductive functions. Network and pathway analysis revealed that the DEPs were predominately associated with posttranslational modification, protein folding and ubiquitination, lipid and nucleic acid metabolism, free-radical scavenging and mitochondrial dysfunction [86]. A total of 73 DEPs were identified by comparing the sperm proteome profile of infertile men with bilateral varicocele against that of fertile men [87]. Most of the DEPs were associated with stress responses, metabolic processes, oxidoreductase activity, enzyme regulation, and immune system processes. Seven DEPs (ODF2, Outer dense fiber protein 2; TEKT3, Tektin-3; TCP11, T-complex protein 11 homolog; TGM4, Protein-glutamine gamma-glutamyl transferase 4; CLGN, Calmegin; TOMM22, mitochondrial import receptor subunit TOM22 homolog; APOA1, Apolipoprotein A-I) were involved in spermatogenesis and sperm functions such as capacitation, motility and sperm-zona binding [87]. Another recent study investigated the proteomic signature of sperm mitochondria in varicocele subjects and identified 25 DEPs related to mitochondrial structure and function as well as core enzymes of carbohydrate and lipid metabolism. Furthermore, the analysis revealed decreased expression of proteins (ATPase 1A4, HSPA2, SPA17 and APOA1) that are crucial for sperm functions such as motility, acrosome reaction and fertilization of oocyte. It was also suggested that mitochondrial electron transport chain (ETC) proteins (NDUFS1, UQCRC2 and COX5B) along with testis-specific pyruvate dehydrogenase (PDH) may serve as biomarkers of sperm function in varicocele subjects Figure 2) [18]. Various sperm proteomic studies conducted in varicocele subjects and the key DEPs identified are summarized in Table 1. Varicocele seems to elicit a stress response during spermatogenesis, with potential impact on protein turn over, post translational modifications and mitochondrial ETC, likely leading to impaired sperm function during capacitation and fertilization.

The pathology of varicocele involves hyperthermia and hypoxia affecting testicular functions [91]. HSPs are considered as molecular chaperones that can sense intracellular and extracellular stress in a cell. The largest group of HSP is the HSPA (HSP70) family containing 13 members. Within the HSPA family, HSPA2 was originally identified as a testis specific chaperone, the expression of which is not induced by heat shock [92]. Most of the sperm proteomic studies in varicocele have revealed decreased expression of HSPA2 [18,87,93]. Furthermore, re-expression of HSPA2 at a higher level has been reported after varicocelectomy [94]. HSPA2 are known to play a key role in regulating the expression of sperm surface receptors that mediate sperm-egg recognition [95]. In animal models, targeted gene disruption of HSPA2 resulted in meiosis failure, increased apoptosis and male infertility [96]. These studies support the theory that lack of stress response against testicular stress particularly heat and hypoxia in varicocele leading to compromised sperm production and function.

#### 4.2.2. Protein Profiling in Testicular Cancer

Testicular cancer (TC) is one of the most common malignancies in men of reproductive age and its incidence has been increasing drastically for the past several decades [97]. The American Cancer Society estimated that there would be 9560 new cases and 410 related deaths for TC in 2019. Deterioration in the semen quality has been reported in TC [98]. In addition, cancer therapy has been reported to have detrimental impact on the fertilizing ability of spermatozoa in these subjects. With the advent of omics, a few proteomic studies have been conducted using testicular tissue samples to identify potential markers for early diagnosis and monitoring of TC [99,100,101]. Recently, Milardi et al. reported that tumor markers AFP, hCG, and LDH can be used in the clinical management of testicular germ cell tumors [102].

Until now, only our lab has investigated the sperm proteomic profile in TC subjects [16,17,25,89,90], and patients with non-seminoma testicular cancer (NSTC) against control fertile men [25]. Furthermore, underexpression of proteins involved in mitochondrial function (NADH: Ubiquinone Oxidoreductase Core Subunit S1;NDUFS1 and ubiquinol-cytochrome C reductase core protein 2; UQCRC2), sperm motility (testis-specific sodium/potassium-transporting ATPase subunit alpha-4; ATP1A4; Annexin A2, ANXA2) and fertilization (ATP1A2, acrosin; ACR) were reported [25]. Comparative sperm proteomic analysis between normozoospermic (motility >40%) and asthenozoospermic (motility <40%) TC subjects before the initiation of cancer therapy revealed proteins associated with the binding of zona pellucida (CCT3), mitochondrial function, sperm motility and exosomal pathway to be differentially expressed in TC patients with asthenozoospermia [16]. Underexpression of NDUFS1 involved in mitochondrial function and overexpression of CD63 associated with sperm maturation were reported in both normozoospermic and asthenozoospermic TC patients when compared to normozoospermic infertile men without cancer [17]. Another recent study from our group revealed alteration in the sperm proteome profile of men with TC seminoma when compared to that of healthy fertile men [90]. The DEPs were associated with spermatogenic dysfunction, reduced sperm kinematics and motility, failure in capacitation and fertilization, which thus attributed to the decrease in fertilizing potential of men with TC seminoma [90]. Apart from TC, men with Hodgkin’s disease have also been reported to exhibit alterations in their sperm proteomic profile, which is associated with low sperm quality [103].

#### 4.2.3. Protein Profiling and Assisted Reproductive Technology

Healthy and superior quality sperm are crucial to ensure successful in vitro fertilization (IVF) of ovum. Despite advancements in assisted reproductive technology (ART) techniques, IVF success rates remain lower, and the specific factors associated with negative outcome still remain to be elucidated. Sperm proteins play a vital role in the fertilization steps. Aberrant expression of sperm proteins is evident in unexplained male infertility cases [104,105,106]. The molecular mechanisms associated with sperm functions such as motility, capacitation, acrosomal reaction and fertilization (sperm-oocyte interaction) are reported to be altered in the spermatozoa of IVF failure patients with normozoospermic semen parameters [104]. The dysregulated proteins were related to sexual reproduction, metabolic process, cell growth and/or maintenance, protein metabolism and protein transport [107]. In addition, the proteins involved in chromatin assembly have been reported to be defective in spermatozoa from normozoospermic infertile men with IVF failure [108,109].

Frapsauce et al. using 2D fluorescence DIGE, identified 17 DEPs in spermatozoa of patients with unsuccessful IVF. Furthermore, LR67 and P34H proteins were proposed as potential targets for the prognosis and diagnosis of fertilization failure in IVF [106]. Similarly, McReynolds et al. using the LC-MS/MS technique demonstrated altered sperm proteome in IVF failure cases. Proteins involved in the spermatogenesis process, such as NME5, TSSK2, MYCBP, MYCBPAP, NDRG3, ROPN1L and SPATA24, were underexpressed in spermatozoa, leading to poor blastocyst formation [110]. A recent study by Liu et al. revealed differential expression of 56 sperm proteins in IVF failure cases, compared to IVF successful cases. These DEPs were associated with molecular functions such as reproduction, chromosome organization, and sperm-oocyte interaction [77].

## 5. Sperm Proteomics in Deciphering Cellular Pathways Associated with Male Infertility

Functional analysis (network and pathway analysis) of proteomic data provides a complete picture of the molecular and cellular pathways affected in the male infertility condition. Bioinformatic programs, such as IPA, provide information about the top enriched canonical pathways and the DEPs associated with diseases and biofunctions. DEPs related to: (1) Oxidative stress; (2) mitochondrial dysfunction; (3) spermatogenesis and sperm function; (4) cytoskeleton integrity; (5) sperm DNA damage; (6) energy metabolism; and (7) protein folding and degradation pathways are dysregulated in spermatozoa of infertile men (Table 2).

Seminal oxidative stress is a major cause of male infertility and is prevalent in 80% of idiopathic infertile men [5]. Excess reactive oxygen species (ROS) results in lipid peroxidation of sperm membrane and damages the sperm DNA integrity, thereby compromising normal sperm functions. A global seminal proteomic study had identified Mucin 5B and oligomeric mucus/gel-forming (MUC5B) as potential indicators of oxidative stress-associated lipid peroxidation [111]. Under oxidative stress condition, proteins associated with carbohydrate metabolic pathways, such as gluconeogenesis and glycolysis, and protein modification are compromised in sperm cells [112]. Ayaz et al. demonstrated that proteins related to sperm function such as acrosome reaction and flagellar movement (ACE, HSPA2, RPS27A, MAP3K3 and APP) are altered in the ROS-induced male infertility [113]. Furthermore, stress response and cellular, metabolic and regulatory pathways are dysregulated in the sperm of infertile men with increased levels of ROS [50].

The mitochondria serves as the power house of sperm, and are hence essential for sperm function. Mitochondrial dysfunction and alterations in the expression of ETC proteins are considered as a major cause of varicocele-related infertility [18]. Protein related to energy metabolism (carbohydrate, lipid and amino acid metabolism) are underexpressed in varicocele patients. Additionally, DEPs associated with spermatogenesis and vital sperm functions such as motility, capacitation, acrosome reaction, and sperm-zona binding were dysregulated in varicocele, oligoasthenozoospermia and asthenozoospermia [70,77,85].

Sperm proteomics of asthenozoospermic patients revealed aberrant expression of HSPs, cytoskeletal proteins, and proteins involved with the fibrous sheath as well as energy metabolism [63]. Hashemitabar et al. demonstrated the difference in the proteome of sperm tail between normozoospermia and asthenozoospermia. Keratin, Type II cytoskeletal 1 protein was reported to be absent in asthenozoospermic men [70]. Mostly, the dysregulation of cytoskeletal proteins reduces the motility of spermatozoa. Cytoskeletal actin-B (ACTB) expression was altered in asthenozoospermic sperm [68], which is essential for cell motility, cytokinesis and organelle movement [114]. 

Defective spermatogenesis and sperm dysfunction (motility, capacitation and sperm-oocyte interactions) are considered as the major cause of fertilization failure resulting in male infertility. Proteomic profile of mature and immature spermatozoa showed that the importin, exportin, and ras-related proteins are markers of sperm homeostasis during spermatogenesis process [115]. Additionally, HSP 70 involved in stage-specific and developmentally-regulated spermatogenesis, A-kinase anchor proteins (AKAPs) regulating the sperm maturation processes of motility, capacitation and hyperactivation, and tektins essential for sperm motility are proposed as the markers of the matured spermatozoa. Aberrant expression of these proteins is mainly due to defects in spermatogenesis [115]. Nixon et al. demonstrated that HSPA2 plays a pivotal role in the regulation of molecular mechanism involved in human sperm-egg recognition [116]. Reduced expression of HSPA2 results in impaired sperm-egg recognition and fertilization failure in both IVF and ICSI treatments [116].

Sperm DNA integrity is regulated by the expression and abundance of sperm proteins. Molecular pathways such as fatty acid binding and prostaglandin biosynthesis functions were reported to be enriched in spermatozoa with damaged DNA [117]. Cysteine-rich secretory protein LCCL domain-containing 1 (CRISPLD1), cysteine-rich secretory protein LCCL domain-containing 2 (CRISPLD2) and retinoic acid receptor responder protein 1 (RARRES1) were proposed as biomarkers for low SDF, while, proteasome subunit alpha type-5 protein was considered to be a potential seminal biomarker for high SDF [117].

Proper regulation of protein folding, and degradation mechanisms are essential for a spermatozoon to fertilize the oocyte. Sperm associated 26S proteasome recognizes and degrades the zona pellucida proteins during the fertilization process [118]. Several studies have showed that sperm proteins involved in protein folding and degradation pathways are dysregulated in male infertility conditions [29,36,85,108]. Recently, Dias et al. reported that degradation of misfolded protein mechanism is affected in TC seminoma patients and HSPA2 protein was proposed as the marker of infertility in men with TC [90].

## 6. Conclusions

Sperm proteomic studies have revealed biomarkers that may aid in the evaluation of male fertility potential, to differentiate between various etiologies of infertility, and also help to predict ART outcomes. Introducing these biomarkers in clinical set ups will transform the diagnostic and therapeutic field of male infertility. However, different DEPs have been reported as potential biomarkers for the same pathology by different studies. This could be attributed to the differences in study specific inclusion/exclusion criteria, sample preparation methods, and techniques used for proteomic analysis. These differences limit the comparison of the data presented in different studies and narrow it down to a cohesive biomarker. Even though the currently available proteomic techniques are highly sensitive and efficient, their use in clinical set up for fertility management is still limited. This is mainly due to the involvement of very expensive and sophisticated instruments that require skilled and well-trained technicians. Introduction of new cost-effective devices and easy to operate proteomic techniques may facilitate a wider application of proteomics in the understanding of male infertility.

## 7. Future Perspective

Over the last decade, there has been a tremendous increase in the number of studies conducted on sperm proteomics, which has undoubtedly facilitated the advancement of clinical research. However, identification of a distinct protein as an ideal biomarker is one of the major challenges for prognosis or diagnosis of a specific pathophysiological state. In general, clinical proteomic studies are conducted in a single sample collected per subject and the proteome obtained is presumed to be reflective of the current pathophysiological state under investigation. However, various factors including, but not limited to demographic, genetic and lifestyle factors could profoundly influence the sperm proteome profile. Taking this into consideration, narrowing down to a single putative biomarker for male infertility related conditions remains challenging. However, it is greatly anticipated that proteomic studies could lead to the development of biomarker panels for the diagnosis and treatment of male infertility [74]. Although routine semen analysis identifies clinical scenarios, such as asthenozoospermia and oligozoospermia, they fail to predict the actual fertilizing potential of these subjects as it is directly related to the molecular characteristic of spermatozoa. An in-depth proteomic analysis would shed light on the potential pathogenic mechanisms underlying abnormalities such as asthenozoospermia, oligozoospermia, and others. In the long run, the proteomic findings may help both in the development of strategic drugs for the treatment of these conditions as well as achieving better results in ART. Therefore, extensive research on the sperm proteome will not only expand our understanding of sperm biology and kinematics, but also revolutionize the field of male infertility and its clinical management in the coming years. In the course of the next five years, the research focus should be geared towards identification and clinical validation of a panel of proteins that could serve as biomarkers for specific male infertility associated scenarios. Focused strategies should be developed for translating the proteomics-derived validated biomarkers from bench to bedside, as these biomarker panels would immensely aid the physician in providing better management and care of patients with male infertility issues.

## Figures and Tables

**Table 1:** Key differentially expressed proteins (DEPs) identified by sperm proteomic profiling in various clinical conditions associated with male infertility.

Clinical Condition	Clinical Scenario	Exclusion/Filtering Criteria	Subjects Enrolled	Samples Used for Proteomics	Method	DEPs	Reference(s)
Varicocele	Oligozoospermic patients with varicocele	Systemic illnesses, cryptorchidism, orchitis, epididymitis, urethritis, testicular atrophy, or sexually transmitted diseases, including human immunodeficiency virus. Azoospermia and a sperm concentration <10 million sperm/mL.	20	20	2D PAGE MALDI-TOF/TOF-MS	HSPA5, ATP5D, SOD1, ACPP, CLU, PARK7, KLK3, PIP, SEMG2, SEMG2pre	[83]
	Unilateral varicocele	Systemic illnesses, cryptorchidism, orchitis, epididymitis, urethritis, testicular atrophy, or sexually transmitted diseases, including human immunodeficiency virus, Endtz-positive. Azoospermia and a sperm concentration <10 million sperm/mL.	33	Pooled sample (*n* = 5)	1D PAGE LC-MS/MS	CABYR, AKAP, APOPA1, SEMG1, ACR, SPA17, RSPH1, RSPH9 DNAH17, DLD, GSTM3, TGM4, NPC23, ODF2GPR64, PSMA8, HIST1H2BA, PARK7	[85]
	Unilateral and bilateral varicocele patients	Endtz-positive. Azoospermia and a sperm concentration <10 million sperm/mL.	Unilateral = 33, bilateral = 17	Pooled sample (*n* = 5/each group)	1D PAGE LC-MS/MS	GSTM3, SPANXB1, PARK7, PSMA8, DLD, SEMG1, SEMG2	[86]
	Bilateral varicocele	Azoospermia and a sperm concentration <10 million sperm/mL. Smoker and abnormal body mass index	17	Pooled sample (*n* = 5)	1D PAGE LC-MS/MS	ODF2, TEKT3, TCP11, TGM4, CLGN, TOM22, APOA1	[87]
	Varicocele	Azoospermia and a sperm concentration <10 million sperm/mL.	50	Pooled sample (*n* = 10)	1D PAGE LC-MS/MS	PKAR1A, AK7, CCT6B, HSPA2, ODF2	[88]
	Varicocele	Endtz-positive and sperm concentration less than <10 million sperm/mL. Female factor infertility	50	Pooled sample (*n* = 10)	LC-MS/MS	LETM1, EFHC, MIC60, PGAM5, ISOC2, TOM22, NDFSU1, UQCRC2, COX5B, ATPase1A4, HSPA2, SPA17, APOA1	[18]
Testicular cancer	Testicular cancer	NA	16	16	1D PAGE LC-MS/MS	PSA, PAcP, ZAG, SEMG 1 and 2, AKAP4, DNAH17	[89]
	Non-seminoma testicular cancer	NA	15	Pooled sample (*n* = 3)	1D PAGE LC-MS/MS	NDUFS1, UQCRC2, ATP1A4, ANXA2, ATP1A2, ACR	[25]
	Normozoospermic and asthenozoosperic testicular cancer	NA	Normozoospermic testicular cancer = 20,	Pooled sample (*n* = 20)	1D PAGE LC-MS/MS	CCT3, ATP5A1, UQCRC2, ATP1A4, MMP9	[16]
			asthenozoosperic testicular cancer = 20	Pooled sample (*n* = 11)			
	Normozoospermic and asthenozoosperic testicular cancer	NA	Normozoospermic testicular cancer = 20,	Pooled sample (*n* = 20)	1D PAGE LC-MS/MS	NDUFS1, CD63	[17]
			asthenozoosperic testicular cancer = 20	Pooled sample (*n* = 11)			
	Testicular cancer seminoma	NA	15	Pooled sample (*n* = 3)	1D PAGE LC-MS/MS	HSPA2, ATP1A4, UQCRC2, ACE	[90]
Asthenozoospermia	Rapid motility (grade a) of 0–3% and progressive motility (grade a+b) of 5–20%	NA	8	8	2-DE MALDI-TOF MS	IDH-α, ODF, SEMG1, ARHGDIB, GOT1, PGAM2, TPI1, CA2, GS10, MSS1	[69]
	Progressive motility <25% (grade a) or motile sperm <50% (grades a + b)	NA	20	20	2D PAGE MS	ACTB, ANXA5, COX6B, H2A, PIP, PIPpre, S100A9, CLUpre, DLDpre, FHpre, HSPA2, IMPA1, MPST/ECH1pre, PSMB3, SEMG1pre, TEX12	[68]
	Rapid linear progression <25% (Grade a)	Sexually transmitted diseases including human immunodeficiency virus (HIV),	17	Pooled sample (*n* = 5)	2D PAGE MALDI MS/MS	TPIS, PSMA3, GKP2, HSPA2, OXCT1, TUBB2C, TEKT1	[67]
	Progressive motility <10%	History of long term medication, varicocele and leukocytospermia. Hyperviscous and necrozoospermic samples, viability <70%.	4		Nano UPLC–MS^E^ tandem mass spectrometry	GRP78, GAPDHS, HSP70-2, TUBA4A, TUBA3C, TUBA8, ODF1, AKAP3, AKAP4, ROPN1B, SPANXB, CLU, PIP, ATP5B, ALDOA, ARGDIA	[63]
	Rapid progressive and slow progressive motility ≤30%	History of depression, diabetes, cancer, hypertension, hyperthyroidism, or sexually transmitted diseases. Exposed to environmental stress, including radiation or chemicals, smokers, and with abnormal body mass index.	35	35	2-DE MALDI-TOF/TOF MS	UBB2B, ODF2, AKAP4, KRT1, CLU, COX6B, GAPDS, PHGPx, HSPA2, HSPA9, VDAC2, GSTMu3, ASRGL1, SPANXB	[70]
	Sperm motility <40%	Endtz-positive.	10	10	UPLC-MS	PLXNB2, POTEKP, NIN, PHF3, DYNLL1, PROCA1, FASCIN-3; LRRC37B, PLC	[64]
	Sperm motility <40%	Oligozoospermia, teratozoospermia or leukocytospermia	4	4	2-DE MALDI-TOF MS	LFT, ATP5B, DJ-1, PARK7, ODF, TEKT1, AKAP4, ELSPBP1, PDHB, NDUS1, SUCLA2, SDHA	[65]
	progressive sperm motility ≤32%	HIV positive samples and sexually transmitted diseases. Samples contaminated with blood	70	Pooled sample (*n* = 5)	2D-DIGE MALDI -TOF-MS	TEX40, ATP6V0A2, SERPINB9, PSA	[66]
Globozoospermia	Round-headed acrosomeless sperm	NA	1	1	2D DIGE MALDI-TOF/TOF MS/MS	SAMP1, ODF2, SPANXa/d, TUBA2, TPI1, PIP	[80]

NA: not available, PAGE: polyacrylamide gel electrophoresis, 1D: 1-dimensional, 2D: 2-dimensional, MALDI: matrix-assisted laser desorption/ionization, TOF: time-of-flight, LC-MS/MS: liquid chromatography-tandem mass spectrometry, UPLC: ultra-performance liquid chromatography.

**Table 2:** DEPs involved in the functional pathways implicated in male infertility.

Molecular Pathways	DEPs	Reference
Oxidative stress	HIST1H2BA, MDH2, TGM4, GPX4, GLUL, HSP90B1, HSPA5, ACE, HSPA2, RPS27A, MAP3K3 and APP, PRDX1, AKAP4	[23,112,113]
Energy and metabolism and Mitochondrial dysfunction	PKAR1A, AK7, CCT6B, HSPA2, ODF2, DLD, ATP5D, NDUFS1, UQCRC2, COX5B, PDH, PHGPx, VDAC, COX6B, AKAP4	[18,70,88,119]
Cytoskeleton integrity	ACTB, KRT1, ODF2, TEK1, TEK4, TEK5, TUBB2B, ACTB	[69,70,107]
Protein folding and degradation	HSPA2, CLU, PSMB4, PSMB5, PSMB6, PSMA3	[36,90,107]
Spermatogenesis and sperm function	Importin, Exportin, HSP 70, AKAPs, HSPA2,	[115,116]
Sperm DNA damage	CRISPLD2, CRISPLD2, RARRES1	[117]
